# Injectable hydrogels of newly designed brush biopolymers as sustained drug-delivery vehicle for melanoma treatment

**DOI:** 10.1038/s41392-020-00431-0

**Published:** 2021-02-15

**Authors:** Aparna Shukla, Akhand Pratap Singh, Pralay Maiti

**Affiliations:** School of Materials Science and Technology, Indian Institute of Technology (BHU), Varanasi, 221005 India

**Keywords:** Cancer therapy, Therapeutics

## Abstract

Novel biocompatible and brush copolymers have been developed for cancer treatment using its controlled drug-release potential. Polyurethane graft on linear dextrin has been synthesized to control the hydrophilic–hydrophobic balance for regulated drug delivery. The properties of the graft copolymers have been tuned through graft density. The prepared grafts are thermally stable and mechanically strong. An injectable hydrogel has been developed by embedding the drug-loaded brush copolymers in methyl cellulose to better control the release for a prolonged period, importantly by keeping the drug release at a constant rate. Cellular studies indicate the biocompatible nature of the brush copolymers whose controlled and slow release of drug exhibit significant cytotoxic effects on cancer cells. Endocytosis of drug tagged contrast agent indicates greater transport of biologically active material inside cell as observed through cellular uptake studies. In vivo studies on melanoma mice exhibit the real efficacy of the controlled drug release from the injectable hydrogel with significant melanoma suppression without any side effects as opposed to severe toxic effects observed in conventional chemotherapy. Special application method of drug-loaded hydrogel just beneath the tumor makes this system incredibly effective through confinement. Thus, brush copolymer injectable hydrogel is a promising vehicle for control release of drug for cancer treatment in future.

## Introduction

Cancer is one of the deadly diseases with high mortality rate across the globe and naturally receives attention since the last few decades. Research thrust is undergoing for the treatment of this dreaded disease. Established approaches for dealing with this chronic disease include surgery, chemotherapy, and radiotherapy, which have certain limitations.^1,2^ Chemotherapy is one of the widely used techniques for cancer treatment, where the killing of cancer cells or suppressing their growth rate is achieved along with serious drawbacks such as destruction of normal cells, and compromised immune system which ultimately leads to the adverse side effects like loss of appetite and nausea etc. One of the important reasons for such high mortality rate is the side effects caused by cancerous drugs over normal tissues and organs.^3^ Major obstacle in chemotherapy lies in non-specificity of drugs which also affects non-malignant normal tissues during the course of treatment.^4^ In particular, targeted delivery systems in cancer therapy are highly desirable to enhance the therapeutic and diagnostic efficacy with minimal side effect.^5,6^ Controlled drug-release systems are being developed to overcome the drawbacks of conventional drug-delivery systems by releasing the drug in a controlled manner at a definite rate in response to stimuli or time, thereby, maintaining the drug concentration in blood stream for longer time and improving bioavailability, its solubility and masking its odor, etc.^7^ Several drug-delivery systems such as particulate carriers^8^, e.g., polymeric gels,^9,10^ and lipids^11,12^ have been used as drug delivery matrices.^13^ The greatest challenge in drug-delivery applications is to develop chemically linked graft copolymers or hydrogels that are biocompatible having higher efficiency, better mechanical strength, swelling property that makes it a better bioadhesive material. Various types of drug carriers have already been reported; among those, the polymers are the simplest where the drug is dispersed within the polymer network.^14^ Porous polymeric hydrogels have been used extensively as carriers for sustained/slow drug release, where the rate of release is controlled through diffusion process or by the combination of diffusion and erosion of the matrix.^15–17^ Recently, the use of natural polymers over synthetic polymers has attained a significant importance for drug-delivery applications, because of their biodegradability, cost effectiveness, and non-toxicity. However, there are some limitations with natural polymers like uncontrolled hydration rate, microbial contamination, and viscosity drop during storage.^18^ Starch-based materials are currently being used to prepare biodegradable hydrogels because of its biocompatibility and degradability with applications in the areas like pharmacy, biology, and medicine. Dextrins are linear *α*-(1,4)-linked D-glucose polymers obtained through enzymatic hydrolysis of corn starch. They also contain 1,6 links (<5%) thus, show branching as well. Dextrin and starch have the same molecular formula [C_x_(H_2_O)_x−1_)]^n−^, where glucose units are linked to each other. Dextrin is small and less complex molecule than starch and is one of the promising candidates commonly used in the manufacturing of adhesives for envelopes gum tapes, postage stamps, and water labeling due to its low viscosity and solution stability at higher temperature.^19^ Diversification of dextrin by grafting or crosslinking is an effective approach that derives the benefit of combining a natural polysaccharide and a synthetic polymer for different biomedical applications including protein delivery,^20^ MRI agent, superabsorbent materials,^21^ as a cross-linker,^22^ and drug delivery.^23^ Grafting of synthetic polymers on natural polymeric backbone is a versatile way to develop polymers with improved functional properties and a suitable drug carrier as well. The composition and structure of the polymers play a significant role in tuning the release rate of drug through controlled diffusion.^13^ Chemical grafting is one of the effective methods for modifying structure and properties of natural polymers.^24^ Graft copolymerization of natural polysaccharides is becoming an important resource for developing advanced materials as it can improve the functional properties of natural polysaccharides.^25^ In this sense, a new generation of graft copolymers combining-starch derivatives and methyl methacrylate (MMA) polymers has been introduced as direct compression excipients. These materials form under compression can control the release of model drug (anhydrous theophylline) through diffusion mechanism from the porous matrix network.^26^ Polyurethanes (PUs) belong to a special class of synthetic polymers owing to biodegradable and biocompatible nature and are currently being used in drug-delivery applications^27–29^ and shape memory properties.^30,31^ These are wonderful polymers with varying molecular designs that can easily be tuned by altering either hard or soft segment, i.e., diisocynate, diol, and chain extenders. Other than traditional applications of PUs, investigations on the development of biodegradable PUs for biomedical applications like temporary scaffold, control release of active ingredients,^32^ and ligament reconstruction prosthesis are under ways. Hydrophilic poly(ethylene oxide) based PU hydrogels have been synthesized and have presented with the desired characteristics that can be used for drug-delivery applications.^33,34^ Grafting of polyurethanes on dextrin can be a new frontier for developing biodegradable and biocompatible polymers, utilizing them as drug-delivery carrier by tuning the hydrophobic wrapping on hydrophilic moiety. Interest lies in investigation of novel chemically linked, biocompatible graft copolymer for sustained drug-delivery applications. In this article, design of polyurethane grafted dextrin copolymers has been done to balance the hydrophobicity and investigate the feasibility of these brush copolymers as a new drug carrier for controlled delivery of anti-cancerous dexamethasone. Developed brush copolymers are characterized using various spectroscopic techniques to confirm their architecture and interactions between brush copolymer and drug molecules. Mechanical and thermal stability of the brush copolymers are worked out to find the suitability of their uses. Drug-release study elucidates the advantage of grafting and the assessment of biocompatibility toward human cervical cancer cells (HeLa) reveals their suitability as drug carrier. The in vivo study on melanoma bearing mice using the novel brush copolymer results in tumor suppression without any noticeable side effect on vital body organs.

## Results and discussion

### Brush polymer of hydrophobic polyurethane on hydrophilic dextrin

**Scheme 1:**
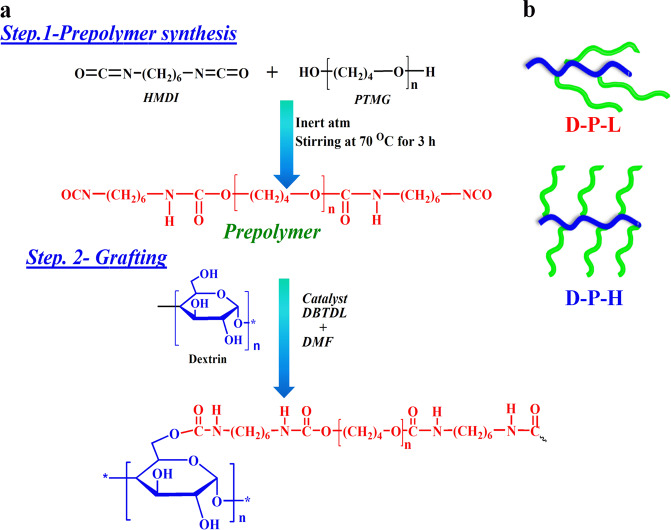
Illustration of prepolymer synthesis and grafting reaction. **a** Reaction mechanism for the synthesis of PU-graft-dextrin with varying degree of substitution. **b** Possible architecture of graft copolymers showing wrapping up in low graft density against relatively open structure in high graft density

**Fig. 1:**
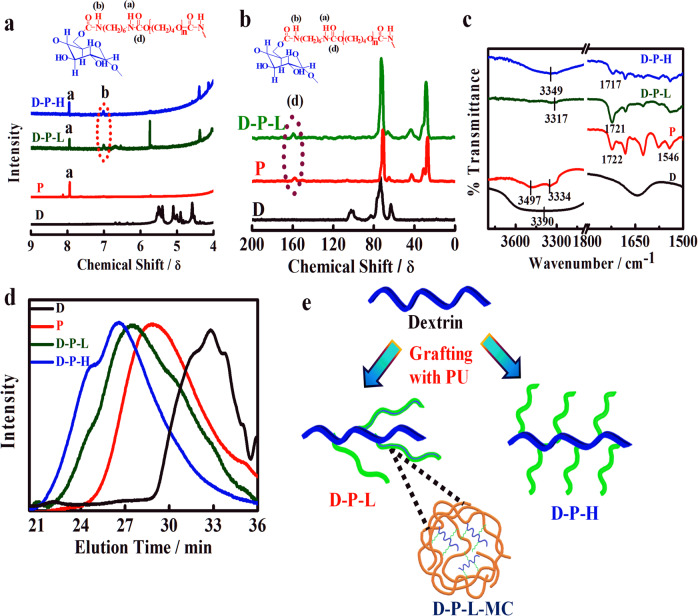
Verification of grafting and interactions between polymeric chains. **a** ^1^H NMR Spectra of linear dextrin (D), polyurethane prepolymer (P) and their indicated grafts showing the change of hydrogen bonding as presented marked by ‘*a*’ and the new peak position due to grafting marked by ‘*b*’ and other NMR peaks information are given in Supplementary information. **b** ^13^C NMR spectra of D, P, and their indicated grafts showing the new peak arising from graft reaction. **c** FTIR spectra of pure dextrin, prepolymer, and their graft copolymers showing shifting in peak positions due to interactions. **d** Gel permeation chromatograms of pure dextrin and its indicated grafts, where high graft density copolymers elute at a lower time. **e** Schematic models showing preparation to two graft density brushes from dextrin and its embedment in methyl cellulose to form injectable hydrogel (D-P-L-MC)

The hydrophilicity of dextrin chains is compromised through grafting of hydrophobic polyurethane with varying the degree of grafting. The general reaction for synthesizing prepolymer and subsequent grafting is shown in Scheme 1 to maintain the hydrophilic and hydrophobic balance. Grafting of polyurethane chain on linear dextrin backbone occurs through the chemical reaction between isocyanate-terminated prepolymer and the hydroxyl group of dextrin forming a urethane linkage (−NHCOO−). Different graft density is achieved by varying prepolymer content over the linear dextrin, therefore, the developed grafts are identified as brush polymers with dense and sparse distribution of hydrophobic side chains. Grafting is confirmed through ^1^H NMR spectroscopy from the peak of urethane proton in prepolymer at 8 ppm, marked as *a* in Fig. 1a, while the appearance of a new signal at 7.0 ppm, labeled as *b*, is due to urethane linkage (−NHCOO−), formed by the reaction of hydroxyl (−ΟΗ) group of dextrin with −NCO (isocyanate) group of prepolymer. The degree of grafting is calculated from the integrated peak area under the peaks and is found to be 15% for D-P-L (low graft density) to 30% in D-P-H (high graft density).^34^ The peaks between *δ* = 4.46 and 4.67 ppm are attributed to protons at positions 2, 3, 4, 5, and 6 (H^2^ to H^6^) while the peak at 5.15 ppm is due to anomeric protons. The peaks indicate the existence of hydroxyl protons –OH (2) and –OH (3, 4, 6), respectively. The peaks at *δ* = 4.538 and from *δ* = 4.863–5.059 represent OH-2, OH-3, and OH-6, respectively, as presented in Supplementary Fig. S1*.*^35^ Since, dextrin contains three hydroxyl groups; two secondary and one primary, the reactivity of primary alcohol is higher, thus, the chemical reaction with isocyanate-terminated prepolymer is assumed to occur with C6 (–OH group) forming the urethane linkage.

Here, it is worthy to mention that the formation of a physical mixture of dextrin and polyurethane is ruled out as NMR spectra of the physical mixture do not exhibit any peak at 7 ppm (Supplementary Fig. S1). The appearance of urethane carbonyl carbon peak for D-P-L (low density graft copolymer) at 157 ppm in ^13^C NMR further corroborates the grafting of polyurethane prepolymer with linear dextrin (Fig. 1b).

A strongly hydrogen-bonded NH bending peak in FTIR spectra at 1546 cm^−1^ is observed in prepolymer whose intensity decreases gradually with increasing graft density signifying insufficient intermolecular hydrogen bonding due to grafting with linear dextrin (Fig. 1c). A shifting in carbonyl peak of urethane linkages at 1722–1717 cm^−1^ in brush copolymers is presumably due to interaction with neighboring polar NH groups.^33^ Pure dextrin shows a broad peak in the range of 3000–3600 cm^−1^ due to multiple hydroxyl groups which become narrower as well as shifted to lower wavenumber upon grafting.^36^ Two peaks in prepolymer occur at 3497 and 3334 cm^−1^ due to free and hydrogen-bonded >N–H stretching, respectively, while these peaks are shifted to 3317 and 3349 cm^−1^ in D-P-L and D-P-H, respectively, indicating intermolecular hydrogen bonding in brush copolymers. Grafting of polyurethane prepolymer on linear dextrin should increase the hydrodynamic volume of the individual polymers and a much higher molecular weight, as measured using gel permeation chromatography, of 83k for D-P-H as compared to 23k molecular weight of D-P-L with a relatively lower and similar polydispersity index of 1.35 for both the samples (Fig. 1d). A bimodal distribution of molecular weight especially for high graft density brush (D-P-H) arises due to mild crosslinking in addition to normal graft reaction. This is to mention that the molecular weight of the physical mixture is almost similar to prepolymer which supports the grafting reaction of prepolymer onto linear dextrin (Supplementary Fig. S2)*.* However, differe nt architectures of brush copolymers are evident depending on the extent of graft density as shown in a cartoon in Fig. 1e, where the brush polymer is embedded further using a gelling agent like methyl cellulose to form injectable hydrogel encapsulating drug molecules within the brush polymer and may act as biomaterial carrier for biologically active agents by altering the hydrophilic–hydrophobic balance. Taken together the NMR, FTIR, and GPC results demonstrate that the chemical linkages between dextrin and polyurethane, i.e., grafting has occurred, which causes higher molecular weight brush with strongly interactive system under varying graft density.

### Microstructure and stability of brush copolymers

**Fig. 2:**
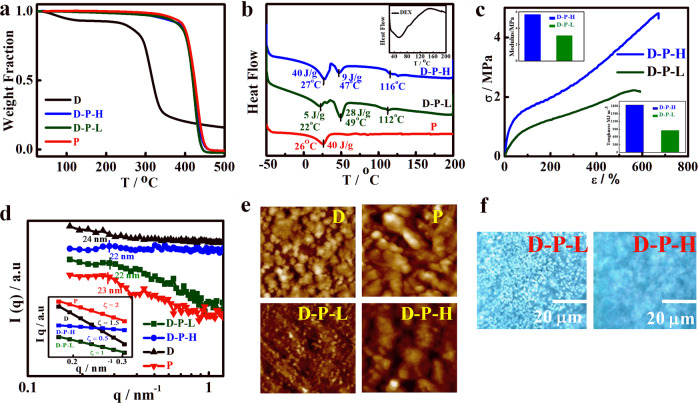
Thermal, mechanical and morphological behavior of brush copolymer. **a** TGA thermograms of pure dextrin, prepolymer (P), and their grafts D-P-L and D-P-H showing their relative thermal stability. **b** DSC thermograms of P, D-P-L, and D-P-H indicating the melting temperature and heat of fusion values. The inset figure represents the melting pattern of pristine dextrin. **c** Stress–strain curves of two different brushes (D-P-L and D-P-H) showing their relative strength. Inset bar graphs indicate the modulus and toughness of the brush copolymers. **d** Small-angle neutron scattering profile of dextrin, prepolymer, and their brushes. Debye–Bueche fittings of the initial data points with correlation length are provided in the inset figure. **e** AFM images of dextrin, prepolymer, and their brushes in semi-contact mode (10 × 10 μm^2^). **f** Optical images of the brush copolymers showing greater agglomerates

Thermal behavior of pure dextrin and its brush copolymers is measured using TGA and DSC to understand the stability and nature of interactions. Degradation temperature is considered as the index of thermal stability and very high degradation temperature of ~350 °C is observed for brushes as compared to pure linear dextrin (230 °C) (Fig. 2a). The thermogram of pure dextrin reveals a two-stage weight loss, the initial weight loss corresponds to the traces of moisture in the sample in the range of 80–130 °C, while the second zone at ~230 °C is due to complete degradation of polysaccharide backbone including primary as well as secondary alcohol groups. Polyurethane grafting provides a hydrophobic character (no initial weight loss) together with high thermal stability through wrapping of high thermally stable polyurethane (*T*_d_ ~ 374 °C, considering 5% weight loss as degradation temperature). However, more than 100° enhancement of degradation temperature occurs in brush copolymer with respect to linear dextrin because of wrapping of thermally stable polyurethane.

Brush copolymers exhibit double melting endotherms with a slight deviation from their two very different constituents. Polyurethane prepolymer shows a single endothermic peak at 26 °C due to the melting of soft segment while corresponding melting temperatures are shifted to 27° and 22 °C in case of D-P-H and D-P-L, respectively (Fig. 2b). The initial reduction in case of low graft density (D-P-L) is presumably due to better interaction between the components (two individual components can lie over each other) while high graft density brush shows higher melting temperature because of facilitated crystallization of larger number of polyurethane units. The extent of crystallization is further corroborated from the differential heat of fusion of 5 and 40 J.g^−1^ for D-P-L and D-P-H, respectively, against the value of pure prepolymer of 40 J.g^−1^. Another peak at 47 and 49 °C is observed for D-P-H and D-P-L, respectively, which is attributed to the melting of pure dextrin (inset of Fig. 2b) and greater deviation in D-P-H is presumably due to dilution effect. Further, a small endothermic peak at higher temperature in the range of 112–116 °C for brush copolymers is due to the melting hard segment of tagged PU. Therefore, TGA and DSC studies indicate better thermal stability of brush copolymer and stronger interactions are evident in modified copolymers.

Mechanical testing is performed to understand the strength and toughness of graft copolymers under uniaxial stretching. The initial slope of the stress–strain curve, a measure of stiffness, is greater for high graft density brush (D-P-H) and Young’s moduli are found to be 3 and 5 MPa for D-P-L and D-P-H, respectively (Fig. 2c). The steep up-rise of the stress–strain curve and high elongation at break for high density brush (D-P-H) is due to strain-induced hardening arising from the entanglement effect of side chain under stress field. Moreover, the areas under the stress–strain curves, a measure of toughness, are found to be 850 and 1840 MJ.m^−3^ for D-P-L and D-P-H, respectively. This is worthy to mention that both the individual components (prepolymer and dextrin) cannot form a mechanically stable film, suitable for uniaxial testing. Thus, mechanical studies suggest that developed brush polymers possess enhanced mechanical properties due to the existence of multiple hydrogen bonds between urethane linkages (cf. discussed in FTIR section) with attached dextrin molecule along with extensive entanglement arising from grafting.^37^ Thus, stiffness, toughness, and elongation at break can be tuned by varying the graft density on linear dextrin.

For better understanding the microstructure of brush copolymers, small-angle neutron scattering is performed which clearly shows a shoulder at wavevector, *q* in the range of ~0.25–0.30 nm^−1^, corresponding to the characteristic length, Λ_c_ of 24, 23, 22, and 22 for dextrin, prepolymer, D-P-L, and D-P-H, respectively, indicating the lesser number of graft molecules required for brushes to form self-assembly (Fig. 2d). The correlation lengths, ξ as calculated from Debye–Bueche fitting (inset of Fig. 2d) of initial wavevector region are found to be 2, 1.5, 1, and 0.5 nm for prepolymer, dextrin, D-P-L, and D-P-H, respectively, clearly demonstrate the smaller blob size in high graft density brush polymers than that of the pure prepolymer.

Polyurethane is prone to form aggregate through hydrogen bonding in hard and soft segment of the segmented polymer chains while high graft density in brush copolymer makes a smaller blob size due to strongly correlated systems through intra- and intermolecular hydrogen bonding as discussed earlier. These crystallite assemblies are further agglomerated to form a strip-like morphology as evident in D-P-H of dimension 900 nm against the usual oval-shaped dextrin dimension of 480 nm (Fig. 2e). Better contrast of segmented zone in D-P-H, almost similar to pure prepolymer, is noticeable as compared to light contrast in D-P-L is primarily due to significant hydrogen bonding in high graft density brush copolymer comparable to the pure prepolymer.^24^ In D-P-H, due to more grafting of prepolymer, the hard domains form self-assembly due to intermolecular hydrogen bonding arising from interchain interactions between >C=O and >N–H groups causing a change in microstructure. Greater inhomogeneities are further noticed from optical images (Fig. 2f) and larger dimensions of agglomerates are observed in D-P-H (5 μm) as compared to D-P-L (2.5 μm). Now, it is important to understand the self-assembly in brush copolymers as evident from the nanometer dimensions of blob size (~2 nm), obtained from SANS, assembled to form 480 nm strip size observed in AFM, which further accumulated micro clusters of 2–5 μm measured from optical images and this systematic self-assembly from nanometer to micrometer size is predominantly through hydrogen bonding originating from polar groups available in linear dextrin and side-chain prepolymer. Therefore, such a bottom-up approach in the construction of self-assembly is most suitable as biomaterial to bind the biologically active molecules and/or drugs that can be released in a sustained manner for better disease control. Moreover, the thermal and mechanical behavior can also be tuned by varying the graft density in brush copolymers, thereby, make them most suitable as drug-delivery vehicle. Surface topology and crystalline structure by using SEM and XRD also support the above discussion and are presented in Supplementary Fig. S3.

### Controlled drug release using brush copolymer and hydrogel

**Fig. 3:**
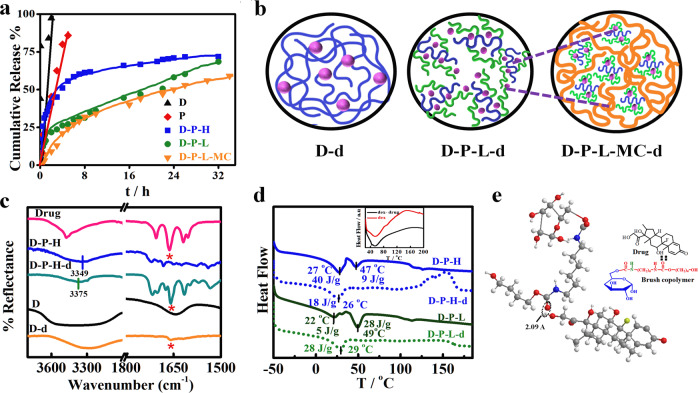
Drug-release profile, release kinetics, and polymer–drug interaction. **a** Cumulative release of dexamethasone from pure dextrin, indicated brush copolymers and its hydrogel showing sustained drug-release profile. **b** Schematic model showing how drug is released from various vehicles. Drug-loaded D-P-L is embedded in MC to make it hydrogel. **c** FTIR spectra of brush copolymers with and without drug loading showing the shifting in peaks as well as appearance of new drug peaks. **d** DSC thermograms of pure and drug-loaded brush polymers showing shift in melting peak along with heat of fusion. Inset figure shows the thermograms of dextrin and drug embedded dextrin. **e** Simulation of energy-minimized configuration of drug and brush copolymer considering one HMDI, one PTMG, and one dextrin unit through semi-empirical AM1 method. The minimum distance between urethane carbonyl of brush copolymer and hydroxyl group in drug is 2.09 signifying hydrogen bonding between the two components

Brush copolymers have been developed which are mechanically strong, thermally stable, and nanostructurally self-assembled and thereby, appropriate material for drug delivery and implant. Sustained drug release is one of the prime concerns to regulate the concentration of drug in the blood stream for a longer time period and to obtain higher efficacy of drug without causing side effects. Cumulative release behavior of a model anti-cancer drug (dexamethasone) from the brush copolymers is shown in Fig. 3a as a function of time comparing with pristine systems. Dextrin and prepolymer release the loaded drug completely within 2 h while the significant sustained but controlled release is observed in brush copolymers and takes nearly a day to release ~50% of the loaded drug. Low graft density brush (D-P-L) exhibits steady release from the beginning (almost ideal behavior) as compared to initial burst release shown by high graft density brush (D-P-H) presumably due to better coverage or wrap up of hydrophobic prepolymer on the hydrophilic chain in (D-P-L) as opposed to stranded pattern of side chain on top of linear dextrin in (D-P-H) as shown in the cartoon in Scheme 1b. As discussed in the previous section, intramolecular hydrogen bonding between polyurethane and attached dextrin in D-P-L is predominant against intermolecular hydrogen bonding in D-P-H leading to open structure through which release of drug is a bit easier in high graft density brush. Moreover, the drug-release rate becomes more sustained, without any burst release, when brush copolymers are embedded in additional gelating agents like methyl cellulose (MC) along with the drug (Fig. 3a). Based on these release profile and interactive nature, a schematic has been designed (Fig. 3b) which depicts the nature of molecular assembly responsible for controlled release using brush copolymer with or without gelating agent. This is worthy to mention that embedment of brush in gelating agent like MC leads to the formation of the injectable hydrogel by controlling the concentration which in turn has enormous application in targeted tumor therapy. However, drug release from delivery vehicle is governed by various factors like, penetration of solvent into the matrix, dissolution of the drug, and finally, the diffusion of drug from the matrix. Amongst these steps, any process can be a rate-determining factor for drug-release kinetics. For understanding the drug-release mechanism, different kinetic models have been used to fit the release pattern from various vehicles. The release kinetics is well fitted with Korsmeyer–Peppas model having higher linear correlation coefficient values with *r*^2^ (~0.98) and having the exponent values of *n* ≥ 0.45 indicating non-Fickian diffusion kinetics of drug molecules except for D-P-H.^38^ Other kinetic models such as zero order, first order, and Higuchi models are presented in the Supplementary Fig. S4 and Table S1. Thus, from in vitro drug-release studies, it is revealed that controlled and slow release of drug is observed which is mainly due to coverage of hydrophobic polyurethane chains onto hydrophilic dextrin backbone along with the tortuous path and interactive nature created by the brush architecture.

The interactive nature of drug and polymers is revealed through spectroscopic and thermal measurements. FTIR characteristic peaks of pure drug at 873 and 1666 cm^−1^ for C–H bending and carbonyl (>C=O) stretching are evident in drug-loaded brush copolymer indicate the presence of the drug in the vehicles (Fig. 3c). Also, there is shifting of the urethane NH stretching peaks in drug-loaded systems toward 3375 from 3345cm^−1^ in pure systems suggest considerable interactions between the components arising from hydrogen bonding and dipole–dipole interactions. There is narrowing of peak in the region of 3300–3400 cm^−1^ in drug-loaded dextrin against broader peak in native dextrin due to interaction of drug with dextrin moiety. Further, lowering of melting temperature in a diluted system is considered as an interactive one and lowering of ~2 °C in drug-loaded system as compared to pure brush copolymers strongly suggest interactive systems (Fig. 3d). Single melting endotherm is evident in drug-loaded system as opposed to double melting for pure brush due to side chain (PU) and main chain of linear dextrin (as discussed before cf. Fig. 2b). Moreover, the significant lowering of heat of fusion also supports the greater interaction between polymer brush and drug. For a better understanding of these interactions, a simulation study of energy-minimized configuration of drug and polymer component (considering one polyol, one diisocyanate, and one chain extender unit) through semi-empirical AM1 method using Chem3D ultra 8.0 is performed. The minimum distance between urethane carbonyl of polymer brush and hydroxyl (–OH) group of drug is 2.09 Å (slightly higher than hydrogen bond) signifying stronger hydrogen bonding between drug and polymer brush (Fig. 3e). Another factor like hydrophobicity of the vehicle may play an important role in drug release as the drug used (dexamethasone) is hydrophobic in nature. The contact angle of D-P-L is found to be 113° against the significant lowering of 87° observed for D-P-H. This is mention that open structure as described earlier in high graft density brush make them porous and/or open structure (cf. Scheme 1b). Hence, the hydrophobic nature of brush along with strong interaction in low density brush is better suited for sustained release of drug over the high density brush. However, control release of drug can be achieved using varying degree of grafting over linear dextrin.

### Biocompatibility and in vitro cell-killing efficiency

**Fig. 4:**
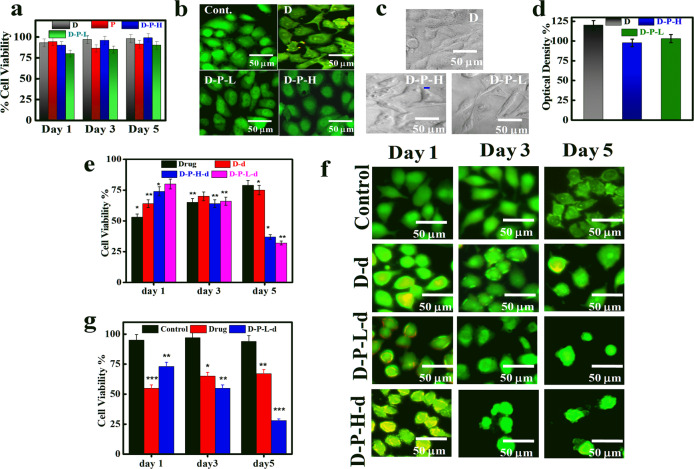
Biocompatibility and in vitro cytotoxicity of brush copolymers and pure polymer. **a** Percentage cell viability obtained through MTT assay measurements of the indicated specimens. **b** Fluorescence images of cells treated with indicated systems after 24 h of incubation. **c** Phase-contrast images of HeLa cells grown on indicated polymeric films, the images of cells adhered on the sample surfaces (cell adhesion). **d** Optical density of the adhered cell for an indication of cell viability. **e** In vitro cytotoxicity by using pure drug and drug-loaded brushes in HeLa cells at 250 μg/ml of drug concentration at different time interval. **f** AO/EB stained fluorescence images of cells after treatment with pure drug and drug-loaded brush copolymers: Results are presented in mean value ± SD, *n* = 5. ***p < 0.001, **p < 0.01, and *p < 0.05. **g** In vitro cytotoxicity potential of pure drug and low graft density brush copolymer (D-P-L at 250 μg/ml of drug concentration) in B16-F10 melanoma cells at different time interval (incubation time)

Biocompatibility of the material is an essential requirement in order to consider it as a good drug-delivery vehicle for biomedical applications and it can be assessed through in vitro cell viability over the surface of the specimen. Viability of HeLa cells over pure dextrin and its brush copolymers is evaluated using MTT assay at different time interval. MTT assay depends on cellular metabolic activity of NADPH enzyme which reduces MTT reagent to purple color formazan crystals in viable cells. Cell viability of brush copolymers is almost comparable to dextrin and slightly lower cell viability of D-P-L vis-a-vis D-P-H is associated with its hydrophobicity as discussed above (Fig. 4a). Therefore, brush copolymers are appropriate as biomaterial either for drug delivery and/or other related biomedical applications. Fluorescence images of the cells after incubation of 24 h are shown in Fig. 4b where the cells are nicely spread and adhered well on polymeric films, further ensure the biocompatibility of these materials. The better proliferation rate and higher cell density over the surface of brush copolymers (D-P-L and D-P-H) even after 5 days of treatment shows their superior biocompatible nature (Supplementary Fig. S5). Cell adhesion studies have also been performed by using HeLa cells showing elliptical morphology of cell over brush surface against the rounded pattern as observed over dextrin (Fig. 4c). Further, the quantification is done through optical density which also confirms the biocompatibility of brush copolymers (Fig. 4d). Thus, the MTT assay concludes that the synthesized brush copolymers possess biocompatible nature, and cells can grow well on these brush surfaces along with their controlled release behavior.

Now, the efficacy of sustained drug release from these brush copolymers can further be explored through comparative cancer cell-killing efficiency. Importantly, the cell-killing efficiency toward HeLa cells increases consistently with time for drug-loaded brush copolymers and reaches up to 70% after 72 h of incubation as opposed to scanty killing of ~25% observed by pure drug and drug embedded in dextrin, considered as conventional vehicle, in the similar time frame (Fig. 4e). The early release of drug from pure drug and drug embedded in dextrin become exposed to cancer cells immediately causing higher killing (~50%) on day 1 and, thereby, the drug is exhausted in the media resulting further increase in cell viability on day 3 and 5, primarily due to natural cell proliferation in absence of drug. On contrary, drug-loaded brush copolymer exhibit high cell viability (low cytotoxicity) on day 1, due to lesser amount of drug available initially, but sustained release of drug with time, killing most of the cells gradually reversing the trend of pure drug and, thereby, avoid frequent dosing as required in conventional delivery system like linear dextrin. Here, it is worthy to mention that poor availability of pure drug for prolonged time (due to its early consumption) could not maintain the effective drug concentration for the cancer cell deaths. Instead, cell proliferation occurs which leads to higher cell viability for a longer time period in case of pure drug and drug embedded in dextrin. Further, the cell viabilities are explored from the fluorescence images of HeLa cells after dual staining with acridine orange and ethidium bromide which clearly indicate the relative cell viability (Fig. 4f) and minimum density of cells are observed for brush copolymer as vehicle as opposed to pure dextrin as vehicle. Both the drug embedded brush copolymers (D-P-H-d and D-P-L-d) show the presence of viable and apoptotic cells. Consistent decrease in cell density is observed with time for brush copolymer as delivery vehicle. Furthermore, brush copolymers show maximum cytotoxicity (70%) in HeLa cells till day 5 and illustrate highest cell growth inhibition vis-a-vis pure drug. Similar cell-killing efficiencies toward melanoma cell line (B16-F10) and ~72% killing is observed in brush copolymer as a vehicle in comparison to meager cell killing (~30%), by pure drug as observed through MTT assay, at the similar dosage and time period (Fig. 4g). Similar observations from fluorescence images of B16-F10 cells treated with pure drug and drug-loaded bush copolymers are observed (Supplementary Fig. S5). The cytotoxicity is directly related to the amount of drug coming out from the vehicle and as usual higher cancer cell death is observed at higher drug doses as presented in Supplementary Fig. S5. In brief, as compared to free drug, a similar amount of drug embedded in brush copolymers exert greater cytotoxic effects. Thus, the efficacy of sustained release is well reflected from cellular studies and the efficacy of sustained drug release from brush copolymers is well demonstrated with a plausible mechanism.

#### Controlled cellular uptake

It is customary that the cellular uptake efficiency of the drug from its vehicles greatly affects the therapeutic efficacy of drug. The drug (dexamethasone) is chemically tagged with contrast agent rhodamine B (Rh) for the cellular uptake studies. Permeation of drug from its surroundings into the cell cytoplasm through cell membrane is essential to understand its consequence toward the cell. Here, drug molecules from the drug carriers are in contact with the cell and its efficacy toward cancer cell death mainly depends on its uptake inside the cell cytoplasm through cell membrane. Hence, a fluorescence agent, rhodamine is tagged with the drug (Rh-D), which does not originally fluoresce, and Rh-D embedded in brush copolymer (D-P-L) matrix termed as GR-Rh-D have been analyzed for their cellular uptake potential. Rhodamine tagged drug can penetrate through cell membrane and can easily be delivered into cell cytoplasm which can be further traced from the fluorescence image of the cells, provided the conjugate pass through the cell membrane.

**Fig. 5:**
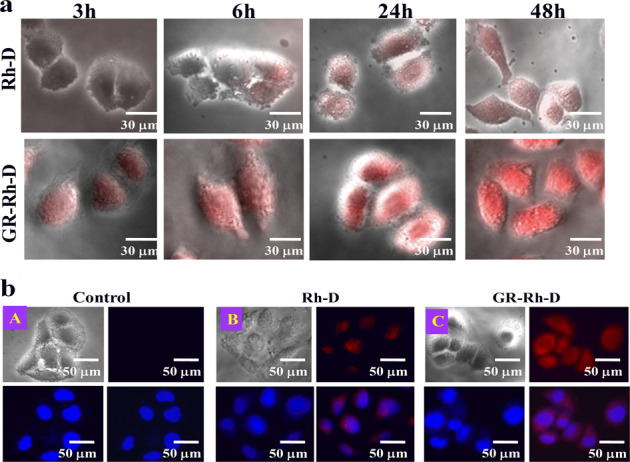
Cellular uptake analysis using rhodamine B. **a** Phase-contrast images of HeLa cells treated with pure drug and drug loaded in brush copolymers after incubation for varying time interval. **b** DAPI stained (blue channel) images of the cell after the treatment with pure drug and drug-loaded brush copolymers. Where the top left is bright field images of cells, top right is rhodamine tagged drug in pure form (red channel) (B) and loaded in brush polymer (C), bottom left denotes cells stained with DAPI (blue channel) and the bottom right is merged image of red and blue channel

Rh-D could not penetrate the cell membrane for the first couple of hours, as evident from no fluorescence up to 5 h of incubation while a certain amount of drug penetrates the cells at 24 h onward (Fig. 5a)*.* In contrast, sufficient fluorescence is noticed from GR-Rh-D at 1 h itself which gradually enhanced as evident from the intense fluorescence image for longer time with uniformity throughout the entire cell cytoplasm. There is a clear differentiation of fluorescence intensity in Rh-D and GR-Rh-D and significantly higher intensity from GR-Rh-D system suggest greater uptake of drug (as the drug is chemically tagged with RhB) using the brush copolymer as the vehicle as compared to pure drug. The greater uptake using brush as the carrier is explained from its better cell adhesion, as observed earlier cf. Fig. 4c, by reducing the negative surface charge after embedment in brush copolymer along with the favorable landing on soft brush, so called better cell adhesion. This is to mention that the high negative zeta potential of dexamethasone restricts its cellular uptake through electrostatic repulsion which gets reduced when it is embedded in neutral brush copolymer, and thereby enhances the cellular uptake of dexamethasone. To understand the location of drug inside the cell, DAPI staining is performed by comparing with control without any treatment. A meager amount of drug has entered into cytoplasm of the cells treated with Rh-D (merge image of red and blue channels of Fig. 5b) while considerable amount of drug has entered into cell cytoplasm along with its nucleus when treated with GR-Rh-D as evident from the merge image of GR-Rh-D (Fig. 5b). Thus, the effect of brush copolymer on cellular uptake is prominent and the efficacy of the brushes as the superior drug-delivery vehicle is demonstrated with mechanistic approach.

### In vivo studies on mice melanoma model

**Fig. 6:**
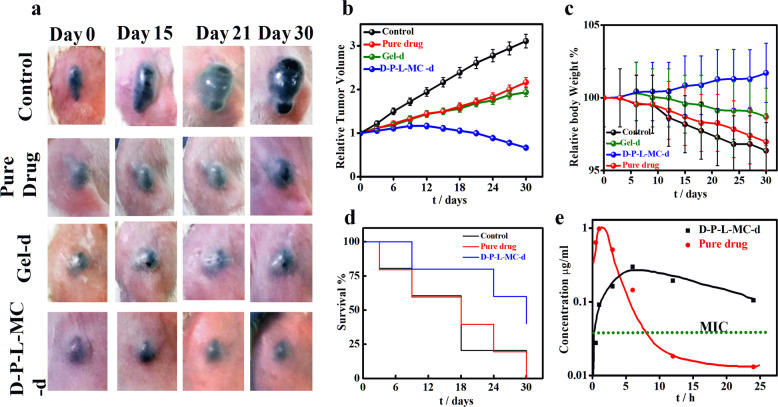
In vivo melanoma studies using mice. **a** Photographic images of mice tumor volume at zero and subsequent days of treatment with *pure drug, Gel-d (durg in MC gel), D-P-L-MC-d* *(drug in brush MC conjugate)*, and control (only saline). **b** Relative changes in tumor volume with time after treatment with different systems as indicated. **c** Relative body weight percentage across the treatment groups. **d** Kaplan–Meier curve of survival percentage of mice after treatment with different systems. **e** Drug concentration profile in plasma as a function of time after intravenous administratrion of durg (5 mg/kg of mice weight) either pure drug and brush conjugated gel (*D-P-L-MC-d*) with equivalent amount of drug. The dotted line indicates the minimum inhibitory concentration (MIC) of dexamethasone

Finally, the efficacy of sustained drug release on mouse melanoma tumor is evaluated using the novel delivery vehicle. Palpable tumors of volume ~20 ± 5 mm^3^ are developed in mice using B16-F10 cell line. Previous literature reports a tumor suppression of 20.6 mm^3^ against 96.4 mm^3^ in control using MPEG-PCL based diblock copolymer loaded with paclitaxel as intra tumoral drug depot.^38^ In order to elude the drawback, the concept of injectable gel is developed with the understanding that the gel when placed subcutaneously will be in close contact with the tumor site for quite some time. Methyl cellulose (MC) is used as gelating agent realizing its gelling behavior and biocompatibility^39^ and can form injectable gel at appropriate concentration. The rheology of methyl cellulose is well reported in literature^40^ and the gelation behavior is performed by inversion test tube method and also through in vivo method shown in Supplementary Fig. S6. Melanoma bearing mice are randomly divided into five groups (*n* = 5) with a subcutaneous injection of only saline (control), brush without drug (*D-P-L-MC)*, pure drug (5 mg/kg), drug in MC (Gel-d), and brush with drug (*D-P-L-MC-d*). It is worth mentioning here that same amount of drug has been used in all the treatment interventions. The tumor volume increases continuously over time for the control (no treatment) while, interestingly, the tumor volume reduces significantly (40% in 30 days) with time for mice treated with *D-P-L-MC-d* gel (Fig. 6a) as compared to other treatment groups.

Tumor volume of mice treated with D-P-L embedded in methyl cellulose gel (*D-P-L-MC*) is found to be similar as that of control in absence of any drug. Pure drug and drug embedded in MC gel (*Gel-d*) reduces the tumor to lesser extent due to immediate and rapid release of drug followed by its reduced or nonavailability afterward as presented in Supplementary Fig. S7. Quantification of relative tumor volume as a function of elapse time is shown in Fig. 6b, indicating the significant reduction in tumor volume in *D-P-L-MC-d-*treated groups while the pure drug or drug embedded in MC gel (*Gel-d*) are unable to reduce the tumor volume to a significant extent as compared to the control. The sustained release of drug from *D-P-L-MC-d* (brush as carrier in gel form) achieves the level of therapeutic dose for a longer period of time (slow and sustained release as evident from Fig. 3a) together with its location, just below the tumor, raises the availability of drug exclusively in the periphery of the tumor. Notably, burst release from pure drug or *Gel-d* exceeds the therapeutic dose within short time, thus, diffuses at higher rate into the blood stream. The tumor growth of mice in pure drug-treated group shows initial inhibition due to burst release but overall inhibition is reduced due to reduced bioavailability in subsequent days. Similarly, only meagre tumor suppression is observed in the mice treated with *Gel-d*. Further, the body weight of mice during experiment show a significant loss in control, *D-P-L-MC* and free drug-treated mice group as compared to *D-P-L-MC-d* where increase of body weight with time indicates the least side effects in the mice (Fig. 6c). Moreover, the mean survival rate, Kaplan–Meier survival plots, illustrates the improved survival percentage of the mice treated with *D-P-L-MC-d* as compared to other groups within the experimental period of 30 days (Fig. 6d). Approximately 40% survival is noticed using *D-P-L-MC-d* as opposed to nearly zero survival in control system. The design of this study is to examine whether *D-P-L-MC-d* can deliver the drug in a sustained fashion by preserving the drug concentration in blood plasma within therapeutic window over an extended time period which can reduce the adverse side effects caused by the fast release of pure drug. To assess and compare the in vivo performance of pure drug and *D-P-L-MC-d*, the concentration of drug in blood stream is measured after an intravenous dose of 5 mg/kg of dexamethasone in two different sets (Fig. 6e). The concentration of drug in blood stream increases abruptly (*C*_Max_ = 1 μg/ml) within 1 h from pure drug system and reach below the MIC just in 8 h post dose. On contrary, gradual increase of drug concentration is observed from *D-P-L-MC-d* system and continues to be higher than the MIC value for more than 24 h clearly indicating the sustained release in animal model as well. This is to mention that the pharmacologically active concentration of dexamethasone is 0.1 μM (equivalent to 39.2 ng/mL).^38^ However, the peak drug concentration in blood for *D-P-L-MC-d* is found to be 0.6 μg/ml and appeared after 6 h of post dose. Further, the *t*_1/2_ for pure drug is ~3.3 h as compared to 11.5 h for *D-P-L-MC-d* which clearly demonstrates the efficacy of sustained drug release from brush copolymer conjugated injectable gel (*D-P-L-MC-d*), thereby, enhancing the residential time of drug in blood stream and increasing its elimination time considerably. However, it is worth mentioning that *D-P-L-MC-d*-treated group of mice shows much higher survival than pure drug-treated group which is consistent with the earlier results discussed above. Thus, the above results indicate that *D-P-L-MC-d* could be a promising carrier for sustained drug release and melanoma treatment.

**Fig. 7:**
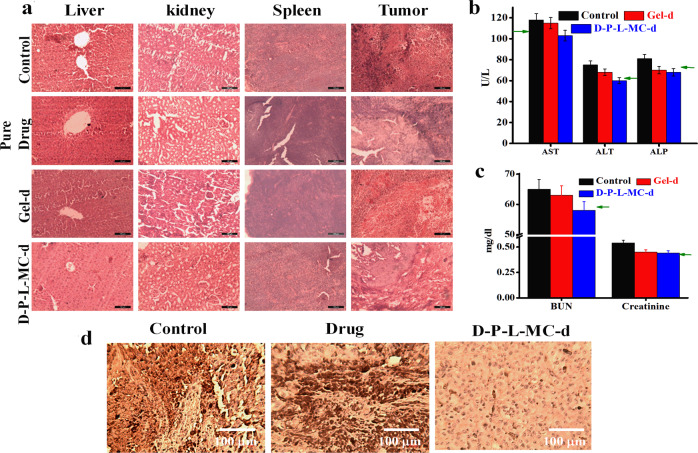
Effect of in vivo controlled drug release and its underlying effects on organs and melanoma tumor. **a** Histopathological analysis of important organs like liver, kidney, spleen, and tumor dissected from mice after 30 days of treatment using H&E staining, at magnification of 10x. **b** Liver function tests by estimating the serum levels of AST, ALT, and ALP across treatment groups. **c** Renal function test by measuring the serum level of BUN and creatinine across the various treatment groups. Corresponding values of healthy mice are indicated by the arrows. **d** Immunihistochemistry of tumor tissues at the end of treatment regimen using melanoma inhibitory activity (MIA) antibody

It is important to know whether the sustained release from the developed carrier does not damage the other body organs while it cures the tumor. For toxicity evaluation on vital organs histopathological analysis is carried out. To assess the toxicity caused by pure drug and drug-loaded systems, hematoxylin and eosin (H&E) staining is performed for important organs, e.g., liver, kidney, spleen, and tumor tissues excised at the end of treatment regimen. The stained images of melanoma, treated with *D-P-L-MC-d* exhibit significant tumor cell death displaying severe necrotic areas with nuclear shrinkages as compared to pure drug and *Gel-d-*treated mice groups (Fig. 7a). Histological architectures endorses the potential of sustained release system using drug embedded in brush (cf. Fig. 6a). Liver histology of pure drug-treated mice exhibits severe damage as evident from inflammation in portal tract and deformation in hepatocytes shape and size while the mice treated with *D-P-L-MC-d* display normal architecture of hepatocytes and also no such toxicity is observed in liver of the mice treated with *D-P-L-MC* and control presumably due to absence of drug. Kidney of mice treated with pure drug and *Gel-d* show tubular injury while in other groups especially in *D-P-L-MC-d-*treated mice, normal kidney architecture is preserved. Spleen in all the systems displays no considerable damage and normal architecture is noticed. Thus, pure drug and *Gel-d* is found to damage the vital organs arising from the burst release of drug, which is a common scenario in conventional chemotherapy for cancer treatment causing mild-to-severe side effect. In contrast, slow and steady release behavior from the developed injectable gel does not cause any adverse side effects on vital organs by maintaining the therapeutic concentration for longer duration. These remedial effects arising from *D-P-L-MC-d* are due to sustained release of drug helps in preserving the bioavailability of drug causing the melanoma shrinkage and overall a better tumor inhibition.

Liver and kidney function tests have been carried out to evaluate the extent of side effects arising from the uptake of pure drug and other drug incorporated systems. Liver enzymes like alanine aminotransferase (ALT) and aspartate aminotransferase (AST) are the critical parameters for the evaluation of proper liver function. AST and ALT values in mice treated with pure drug showed a considerable increase (ALT ∼ 75 U/L and AST ∼ 118 U/L) from the normal values after 30 days of treatment indicating severe liver damage, while the mice group treated with *D-P-L-MC-d* display liver activity similar to normal mice (indicated by the respective arrows) (Fig. 7b), strongly suggest the safe application of *D-P-L-MC-d* in cancer treatment. Similarly, blood urea nitrogen and creatinine levels, indicator of renal health, have been increased in mice treated with *Gel-d*, while reporting the normal range for mice treated with *D-P-L-MC-d* (Fig. 7c). As a consequence, the liver and kidney are severely affected by the administration of pure drug. Further, the immunohistochemistry of tumor tissues stained with melanoma inhibitory activity (MIA), a protein specific to melanoma cells, are performed to understand the level of tumor suppression. MIA is an early indicator of tumor progression relapse and metastasis.^41^ MIA is expressed only on melanoma cells and the extent of inflammations upon treatment can easily be visualized with MIA inhibitory compounds. The expression of this protein is maximum in control group suggesting that highly active melanoma proliferation which has been found to be reduced up to some extent in mice treated with pure drug. Significant and homogenous MIA protein expression is directly correlated with severity of melanoma.^42^ The extremely low expression is observed in mice treated with *D-P-L-MC-d* which indicates the beneficial effect of sustained drug-release system that could suppress MIA activity in melanoma tumors (Fig. 7d).

Design of drug carrier through wrapping of hydrophobic layers through grafting over hydrophilic chains regulates the hydrophilic–hydrophobic balance by altering the extent of grafting to embed lyophobic anti-cancer drug. Ideal drug-delivery behavior is achieved through various interactions and special architecture of the vehicle. Better biocompatibility along with its non-toxic nature help control the tumor progression without any noticeable side effect. This vehicle has every potential to act as drug-delivery carrier especially in cancer treatment as proven.

### Conclusion

Hydrophilic dextrin is grafted with hydrophobic polyurethane to generate brush copolymers with varying architecture of different graft density. Gradual increase of molecular weight together with contact angle indicates the change over from hydrophilic to hydrophobic moiety and complex brush-like structure. Sustained release of drug without any initial burst release has been achieved using brush copolymer as the drug-delivery vehicle following the non-Fickian kinetics. Strong interaction between the drug and brush copolymer together with its complex structure helps improving the sustained drug release suitable for chronic diseases including cancer. Biocompatibility of the brush copolymer is verified through MTT assay and cells can adhere better than those pure components of the brush copolymer. Very high cell-killing efficiency of ~70% is achieved using brush as delivery vehicle against the meager 25% killing using pure drug at similar concentration and the phenomena is explained from the sustained drug-release behavior of brush copolymer as opposed to burst release in pure drug. The uptake of drug using brush copolymer as carrier is found to be superior as compared to pure drug arising from the repulsion of surface charge with negatively charged cell membrane and the cytotoxic efficiency is found to be excellent in different cancer cell lines. The efficacy of sustained release is verified toward tumor suppression in animal model with low mortality rate and increase of body mass index during the treatment period vis-à-vis very high mortality, reduced body weight and only a slight decrease in tumor size is observed using pure drug of similar dosage. Injectable hydrogel has been developed using gelating agent by which gel can be placed just below the tumor site and can release the drug in sustained manner, exclusively to the tumor site. Importantly, the vital body organs are unaffected in the group of mice treated with brush copolymer as drug-delivery vehicle as opposed to severe liver and kidney damaged in mice group treated with pure drug, as often noticed during conventional cancer chemotherapy. The significantly reduced MIA expression confirms the efficacy of novel drug-delivery vehicle as compared to pure drug. Furthermore, MIA study reveals that sustained drug-delivery system enhances the bioavailability of drug for longer time period that suppresses the tumor progression through altered gene expression of cancer cells.

## Materials and methods

Poly(tetramethylene glycol) (PTMG) (terathane, Sigma-Aldrich; number average molecular weight, Mn = 2900 g mol^−1^) was used as received, 1,6-hexamethylene diisocyanate (HMDI) and the catalyst dibutyl tin dilaurate (DBTDL), the solvent dimethyl formamide (DMF) were purchased from Merck, Germany. Dextrin and Rhodamine B were purchased from Himedia and anti-cancer drug dexamethasone was purchased from Sigma-Aldrich, USA.

### Synthesis of polyurethane and brush copolymers

The synthesis of polyurethane was carried out in two consecutive steps; the first step prepolymer formation using soft segment PTMG and HMDI for 3 h at 70 °C yielding isocyanate-terminated prepolymer while the second step involves in chain extension by adding various weight ratios of dextrin in the presence of catalyst DBTDL (0.1 ml of 1 wt% toluene) and DMF as a solvent to carry out the polymerization process with vigorous stirring. The reaction was performed in three neck flask well-equipped with mechanical stirrer and nitrogen purging inlet to maintain inert atmosphere in silicon oil bath keeping the temperature at 70 °C for 24 h. Reaction mixture was poured in deionised water which is a non-solvent for precipitation of the copolymers. Washing was done repeatedly to separate out unreacted dextrin and HMDI. Polymers were dried in air oven at 60 °C for 24 h and in vacuum oven for 24 h. Reaction scheme details for copolymers synthesis is given in Scheme 1. The molecular weight and its distribution determination of synthesised copolymers was done through GPC technique. Polymers are abbreviated accordingly where, D is dextrin, P is the PU prepolymer, and D-P-L and D-P-H are their respective low and high graft density copolymers, respectively. H and L signify the high and low graft density of prepolymer (P) over the dextrin chains.

### Preparation of injectable hydrogels

The synthesized brush copolymer D-P-L was dissolved in DMF and then was embedded in 10 wt% methyl cellulose (MC, as gelating agent) yielding the whole system as injectable gel and is termed as D-P-L-MC. The concentration of MC was so chosen the gelatine time is around 5 min, suitable for injectable gel formation in animal model.

### Characterization

#### Spectroscopic measurements

^I^H NMR spectra of copolymers were recorded in D6 DMSO solvent using Bruker AVANCE 400 MH Spectrometer (Germany). For FTIR analyses, solid polymeric thin films were prepared and recorded on a Thermo Nicolet 5700 instrument with the resolution of 4 cm^−1^ taking 100 scans at room temperature. Measurement was done under reflectance mode in the range from 650 to 4000 cm^−1^. The UV–Vis measurements of transparent thin films prepared through hot pressing were performed by using a Jasco V650s spectrophotometer, Japan, in the range of 200–800 nm.

#### Thermal and mechanical studies

Thermogravimetric (TGA) measurement was done for the estimation of degradation temperature of synthesised copolymers by using thermogravimetric analyzer (TGA, Mettler-Toledo) at a heating rate of 20° min^−1^ under nitrogen atmosphere in the temperature range from 40 to 600 °C. Melting behavior of pure dextrin and its graft copolymers along with drug-loaded polymers was determined through differential scanning calorimetry, Mettler 832. Calibration of DSC was done with indium before use. The quenching of the films occurred from room temperature to −50 °C at the rate of 30 °C/min followed by heating up to 200 °C at the rate of 10°/min. Heat of fusion was calculated from the area under endothermic peak. Mechanical properties of polymers were studied via tensile strength measurement using universal testing machine (Instron 3369) at a strain rate of 5 mm/min at room temperature. Polymeric films were prepared through solution route and specimen having dimensions of 30-mm long, 4-mm wide, and 1.2-mm thick were used for the study. Three films of each specimen were tested to obtain good error estimates through standard deviation.

#### Morphology and structural characterization

XRD measurement of the films, prepared through compression molding technique, was done using a Rigaku MiniFlex Advance wide-angle X-ray diffractometer with a graphite monochromator using CuKα source with a wavelength of 0.154 nm. The samples were scanned at a rate of 3°/min. Small-angle neutron scattering measurements were performed on a spectrometer at the Dhruva reactor in Bhabha Atomic Research Center, Mumbai, India. The scattering range of the wavevector was kept constant for all the samples and data were collected in the range of 0.17 nm^−1^ ≤ *q* ≤ 3.5 nm^−1^. Initial lower scattering wavevector range was fitted with Debye–Buche model. The characteristic length was calculated through the equation Λ_c_ = 2π/*q*_m_, where, *q*_m_ is the scattering wavevector corresponding to the peak position in the scattering pattern. Surface profiles were measured using NT-MDT multimode AFM, Russia, controlled by a Solver scanning probe microscope controller. Semi-contact mode of AFM was used with the tip mounted on 100-mm long, single-beam cantilever with resonant frequency in the range 240–255 kHz, and the corresponding spring constant of 11.5 N/m. Overall bulk morphology of thin films of synthesized brush polymers in optical range was investigated through polarizing optical microscope (Leitz). Three different samples were examined at different sites for elimination of artefacts. SEM analyses of the brush polymers were investigated through a scanning electron microscope (SEM; Supra Zeiss, Germany), by fixing the samples on a metal stub with conducting carbon tape. Gold coating on the samples was done by means of a sputtering apparatus before the observation under SEM. Contact angle was measured with a Kruss tensiometer K-100 at room temperature. Samples in triplicates were used for error estimation during each experiment.

#### Drug assay and release

In vitro drug release for dexamethasone was carried out in phosphate buffer saline at pH ~ 7.4 and 37 °C. Briefly, the standard stock solution of dexamethasone (1 mg/ml) was prepared and standard curve was drawn using UV–Vis spectrophotometer, Jasco V650 at a wavelength of 242 nm in the concentration range from 1 to 100 μg/ml. The known weight of polymer was dissolved in DMF, after complete dissolution of dexamethasone (5 wt% of the polymer) was added and the solution was stirred for 24 h at 60 °C to ensure proper mixing of drug with polymer and its encapsulation inside polymeric matrix completely. Solvent was evaporated by heating at 60 °C to prepare the drug-loaded films. The drug-loaded film was kept in the flask containing 50 ml PBS solution, placed in an incubator shaker at 100 rpm at 37 °C. After regular time interval, a measured amount of solution was removed and was replaced each time with fresh buffer solution of equal amount. Drug amount in each fraction was known by recording the absorbance at 242 nm.

### Biological studies

#### Cell culture and cell adhesion

HeLa and B16-F10 cancer cells were cultured in Dulbecco’s modified Eagle medium (DMEM) containing 10% heat-inactivated fetal bovine serum, 100 μg/ml penicillin, and 100 μg/ml streptomycin. The culture was maintained at 37 °C in a CO_2_ incubator with 5% CO_2_ supply. Cell viability of pristine as well synthesised polymers was analyzed through MTT assay on HeLa cells. Films of each specimen were first sterilized under UV for 3–4 h and then transferred at the bottom of 96-well plate followed by cell seeding at the density of 10^5^ cells. The cells were cultured for 1, 3, and 5 days in the CO_2_ incubator (5%) at 37 °C. All the treatment was done in triplicate for the assay. One hundred microliters of MTT solution prepared in DMEM media 0.5 mg/ml was added to each well after the desired incubation period. One hundred microliters of DMSO was added in each well after 4 h of MTT addition to solubilize the formazan crystals formed. Absorbance was measured with the help of microplate reader at the wavelength of 570 nm. Percentage cell viability was calculated using the following equation;

$${\mathrm{\% }}\;{\mathrm{Cell}}\;{\mathrm{viability}} = \frac{{{\mathrm{OD}}\;{\mathrm{of}}\;S}}{{{\mathrm{OD}}\;{\mathrm{of}}\;C}} \times 100$$

where *S* is the optical density of the test sample and *C* optical density of control (no treatment to HeLa cells).

For the cell adhesion, HeLa cells at the confluence of 1 × 10^4^ per ml were seeded onto polymeric film surface in 12-well plate and incubated for 6 h in CO_2_ incubator. After the incubation period, cells were washed with PBS solution to remove the unattached cells from the samples. Cell fixing was done with 4% paraformaldehyde for 15 min followed by washing with PBS again. After that, cell permeabilization was done with 20% methanol for 20 min. Staining of adhered cells was done by using 0.2% crystal violet aqueous solution for 15 min. Washing with PBS and 10% acetic acid was done to remove excess stains and to remove the residual crystal violet. Optical density of solution was measured using microplate reader at a wavelength of 570 nm. Cell adhesion images were also captured using a phase-contrast microscope (Leica, Germany) after fixing the cells with 4% paraformaldehyde solution followed by washing with PBS. Percentage cell adhesion was calculated using the following formula:

$${\mathrm{\% }}\;{\mathrm{Cell}}\;{\mathrm{adhesion}} = \frac{{{\mathrm{OD}}\;{\mathrm{of}}\;S}}{{{\mathrm{OD}}\;{\mathrm{of}}\;C}}\times100$$

#### Fluorescence imaging

Cells were grown on polymeric films similar to cell viability studies. After 24 h of incubation, cells were washed with PBS solution twice to remove dead and unattached cells, fixed with 4% paraformaldehyde for 15 min followed by staining with fluorescent dye acridine orange and ethidium dibromide (0.1 mg/ ml) for 10 min. Excess stain was removed by PBS washing and images were taken using fluorescent microscope (Leica, Germany).

#### Cellular uptake

Pure drug was labeled with the dye Rhodamine B (RhB). First, the drug was dispersed in DMSO to which a predetermined amount of RhB in water was added. The reaction was carried overnight at room temperature with vigorous stirring in dark. After 16 h RhB labeled drug abbreviated as Rh-D was washed thoroughly with water and the solution was centrifuged and the tagged drug was collected, dried in an oven at 50 °C. Further, Rh-D was embedded in polymer through solution route, by dissolving the polymer in DMF solution and by adding Rh-D dispersed in water (5% of Rh-D w/w% w.r.t. to D-P-L). This prepared solution was stirred overnight to ensure proper mixing of Rh-D and polymer. The solution was poured into petridish for solvent evaporation for 24 h and then vacuum dried for another 24 h. This was designated as GR-Rh-D. In short, 1 × 10^4^ HeLa cells were cultured in a 24-well plate for 24 h. Cells were treated with Rh-D and GR-Rh-D and were incubated for 3, 6, 12, and 48 h for understanding the GR-Rh-D uptake process as a function of time. The debris or extracellular materials was washed using PBS solution and the images were captured by using an inverted fluorescence microscope. 4′,6-diamidino-2-phenylindole (DAPI) staining was done by using 30 μM DAPI solution which was incubated at room temperature for 15 min and was washed thrice with PBS solution. The cellular imaging was performed using an inverted fluorescence microscope (Leica, Germany).

#### Anti-tumor efficacy of drug loaded in polymer brush

Subcutaneous tumors were accomplished by inoculating 1 × 10^6^ B16-F10 cells at the right flank of mice (6–7 weeks old, supplied by the Institute of Medical Sciences, Banaras Hindu University, India) and were allowed to acclimatize for 7 days. When the tumor volume reached to 20 ± 5 mm^3^, mice (*n* = 5 per group) were injected subcutaneously at the tumor site with saline (control), brush polymer gel (*D-P-L-MC)*, pure drug (d) (5 mg/kg), drug in MC gel (Gel-d) (5 mg/kg), and 5 mg/kg of drug-loaded brush polymer gel (*D-P-L-MC-d*) once in every 3 days for 30 days. Tumor volume was measured on every third day with the help of vernier calliper and volume measurement was done according to the equation; tumor volume = (length × width^2^)/2. The tumor volume, body weight, and mortality rate were monitored for all the experimental groups as a function of time.

#### Biodistribution study

For the biodistribution study, blood was collected at 0.5, 1, 3, 6, 12, and 24 h after the treatment with pure drug and *D-P-L-MC-d* gel at an equivalent dose of 5 mg/kg via tail vein injection. Immediately after blood collection, blood was centrifuged at 4000 rpm for 10 min at 4 °C to obtain the required serum. The mobile phase consisted of a mixture of acetonitrile and water (60:40 v/v) freshly prepared for each run. Twenty microliters of each sample was injected into the column for analysis. The mobile phase flow rate was kept at 1.0 ml/min and was detected at a wavelength of 242 nm.

#### Histopathology and biochemical analysis

For histological analyses, mice were sacrificed at the end of the experiment, and vital organs like liver, kidney, spleen, and tumor tissues were excised for further study. Further, the dissected organs and tissues were preserved in 7% formalin solution for 48 h. Dehydration was done in graded ethanol and tissues were embedded in paraffin wax. The tissues were sliced into sections of 3–5 µm thickness and were stained using H&E. The stained slides of these sections were then examined under light microscope at a magnification of ×10. For biochemical analysis, blood serum was collected (50 μl/mice) at the end of treatment for the clinical assessment of blood parameters like aspartate aminotransferase (AST), alanine aminotransferase (ALT), alkaline phosphatase (ALP), blood urea nitrogen (BUN), and creatinine.

#### Immunohistochemistry

Slides were deparaffinised by using fresh xylene for 5 min followed by washing with running water for 5 min. For quenching endogenous peroxidase activity slides were placed in 3% H_2_O_2_ solution for 10 min, sections were washed with distilled water for 5 min, each followed by washing with tris buffer solution (TBS) for 5 min. Then, the slides were kept in a preheated jar for 5 min. After that, washing was done with tap water and phosphate buffer solution. The section was marked and protein blocking was applied on section for 15 min at room temperature. Blocking solution was removed and treatment with 25 μl of primary antibody, MIA (sc-377375 Santa Cruz Biotechnology, Inc.) at the dilution of 1:100. Slide was kept in petridish which was placed in 4 °C freezer overnight for antibody stain. Slide was washed with TBS, secondary antibodies were added and incubated for 15 min followed by washing with PBS. DAB was added on slide for 1 min, washed with water, and counter stained with hematoxylin for 1 min. Slides were dried and mounting was done with DPX. Images were captured using optical microscope (Leica, Germany).

#### Statistical analysis

Data were expressed as mean ± standard deviation (SD). Difference between groups was assessed using one-way ANOVA with Tukey tests, wherein *p < 0.05 was considered significant, and **p < 0.01 and ***p < 0.001 were highly significant.

## Supplementary information

Supplementary information

## Data Availability

The data sets used for the current study are available from the corresponding author upon reasonable request.
