# Supplementary material for: Injectable hydrogels of newly designed brush biopolymers as sustained drug-delivery vehicle for melanoma treatment
Source: Signal Transduct Target Ther. 2021 Feb 15;6:63. doi: 10.1038/s41392-020-00431-0 (PMC7884735; doi:10.1038/s41392-020-00431-0)
Supplement: Supplementary file 1 — Supplementary information [file 41392_2020_431_MOESM1_ESM.docx]

Supplementary Materials for

**Injectable Hydrogels of Newly Designed Brush Biopolymers as Sustained Drug Delivery Vehicle for Melanoma Treatment**

Aparna Shukla , Akhand Pratap Singh, and Pralay Maiti

Correspondence to: [pmaiti.mst@itbhu.ac.in](mailto:pmaiti.mst@itbhu.ac.in).

Corresponding author: Prof. Pralay Maiti, Professor, School of Materials Science and Technology, IIT BHU, India; Phone: +91 9935141321; Email: [pmaiti.mst@itbhu.ac.in](mailto:pmaiti.mst@itbhu.ac.in).

**This PDF file includes:**

Figures S1 to S7

Tables S1

**Material and method**

**Cellular uptake**

Drug dexamethasone was labeled with rhodamie B (RhB) by following approach, first the known amount of drug was dispersed in 10 mL water. A definite amount of RhB was added in water and this solution was added to drug dispersions dropwise. The solution was stirred overnight at room temperature in dark. After completion of the reaction, RhB labeled drug was washed several times with ethanol and deionized water, till water became colorless to ensure complete removal of free RhB. Rhodamine being a hydrophobic fluorescent dye, was efficiently tagged with hydrophobic drug molecules. RdB-labeled drug are abbreviated as Rh-D.

Probable site for connections between drug and rhodamine molecules is between carboxylic group of dye and primary hydroxyl group of drug (dexamethasone).

**Figure. S1.**


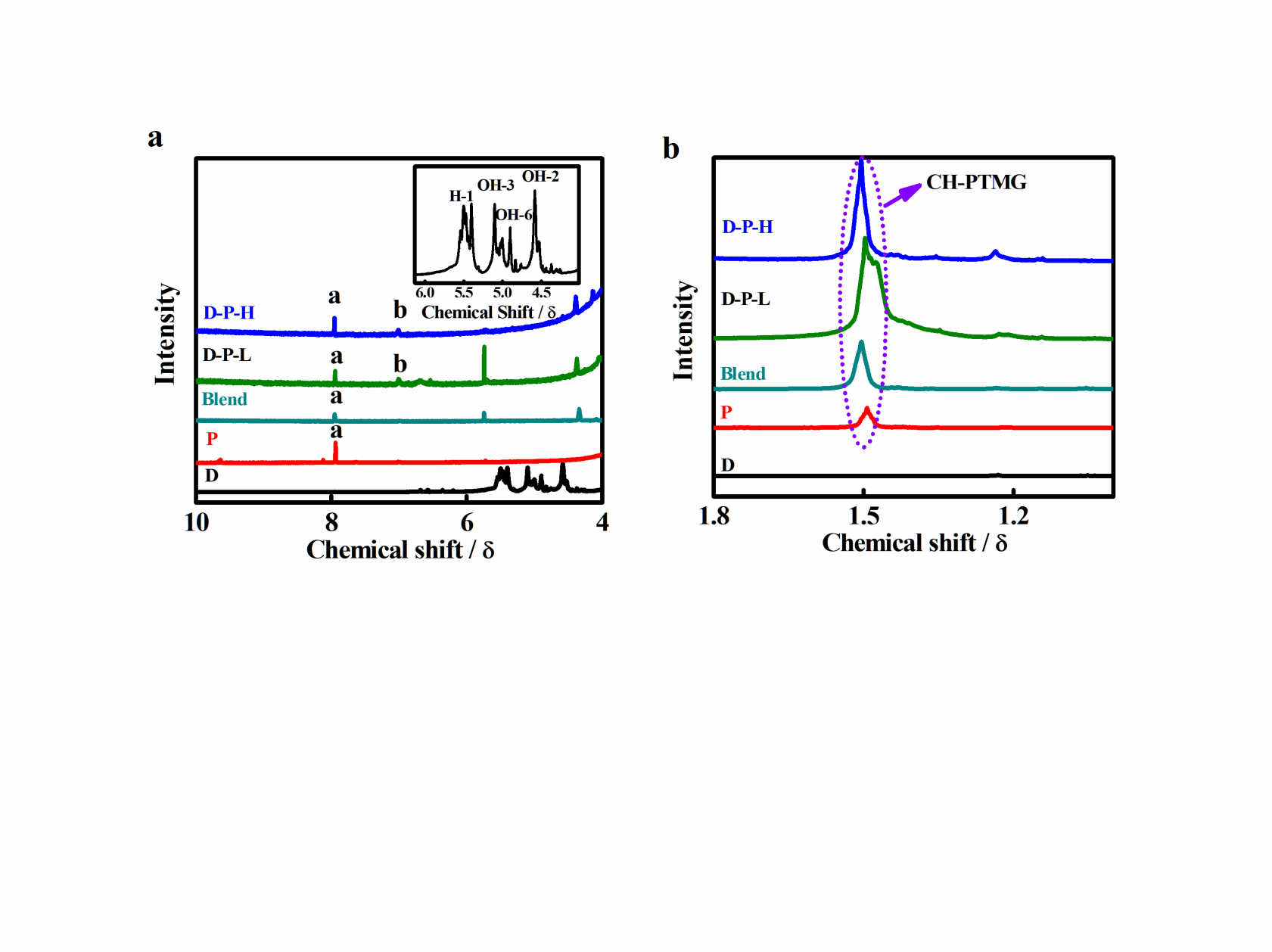


**Figure S1: a)** Proton NMR of all the copolymers absence of peak at 7 ppm in blend proves the chemical connection of dextrin with polyurethane. All other respective peaks of dextrin are well matched with literature and reported. **b)** NMR spectra showing new peak at 1.5 ppm in all copolymers signifying CH of PTMG due to the grafting of polyurethane.

**Figure. S2.**

**Figure S2:** GPC chromatogram showing molecular weight of physical mixture (blend) is almost similar to pure prepolymer confirming that it’s a physical mixture while in graft copolymers there is chemical connections reflected from their respective higher molecular weight.

**(c)**

**Figure. S3.**


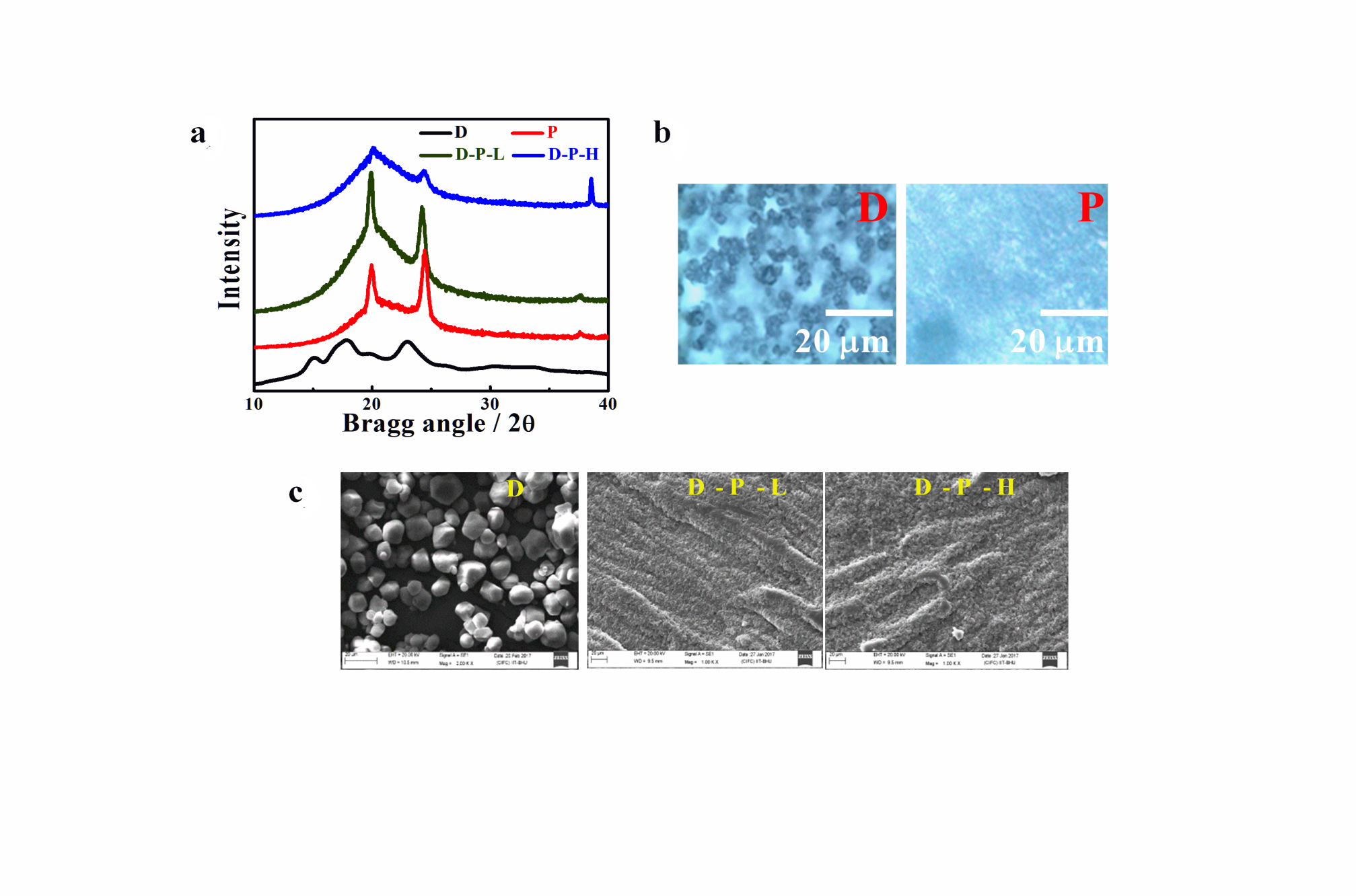


**Figure S3: a)** XRD patterns of pure Dextrin, P and their graft copolymers; **b)** optical images of the dextrin and prepolymer showing greater agglomerates and **c)** SEM images of dextrin showing granular morphology and its graft copolymer.

**Figure. S4.**


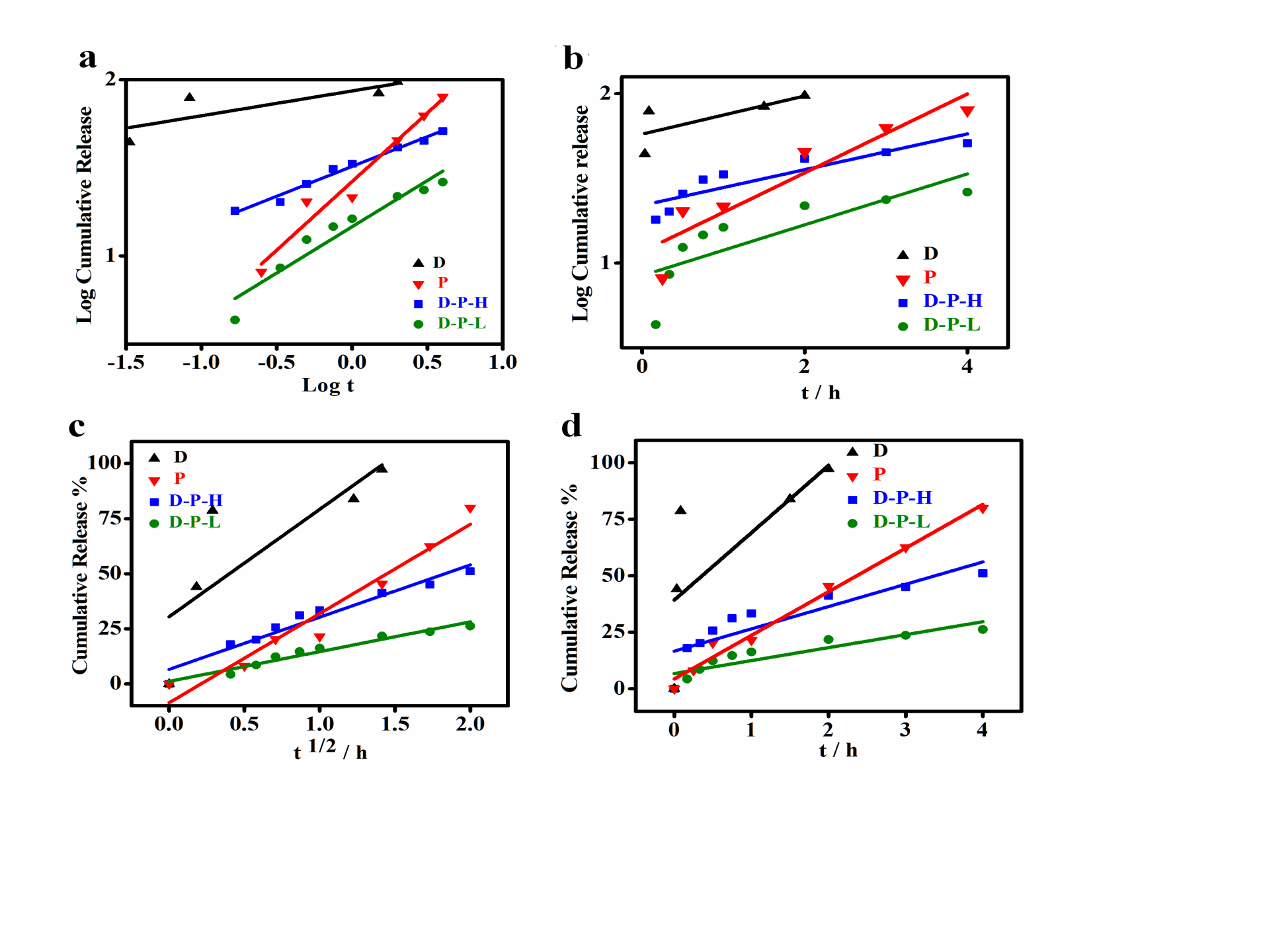


**Figure S4:** Mathematical models for drug release kinetics. (a) Korsmeyer- Peppas model, (b) First order model, (c) Higuchi model, (d) Zero-order model. The correlation coefficients along with other parameters for each model are presented in the table below.

**(c)**

**Table. S1.**

**Table S1:** Release rate constant (k), correlation coefficient (r^2^) and diffusion release
 exponent (n) obtained using different mathematical models for drug-loaded D and
 its respective copolymers.

**Figure. S5.**


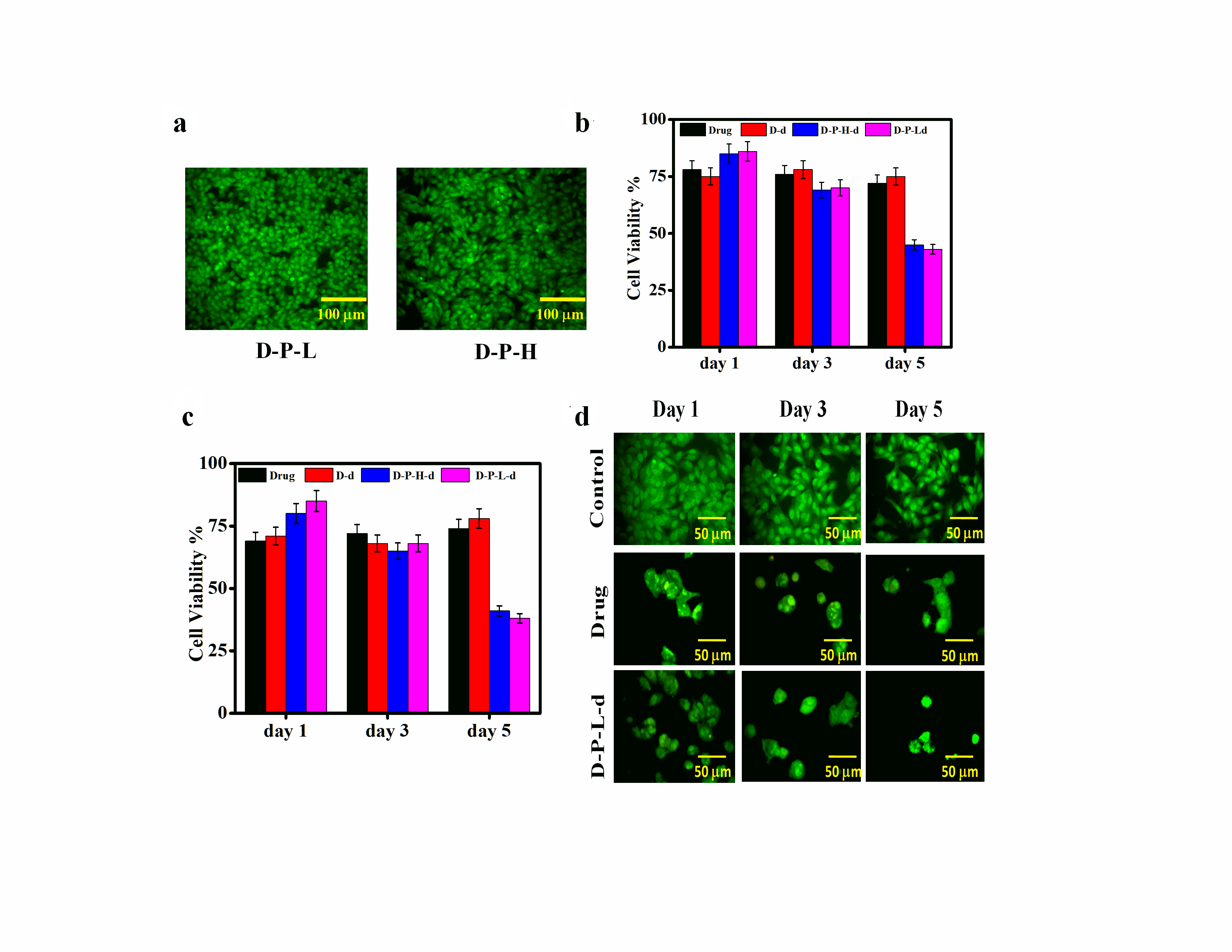


**Figure S5: Biocompatibility and dose dependent cytotoxicity of brush copolymers after different time interval**. a) Fluorescence images of brush copolymers after 5 days of treatment showing colony of cells indicating the biocompatibility of brush polymers. Cell viability of HeLa cells after incubation of drug and drug-loaded different brush polymers as indicated, (b) concentration range of 20 μg/ml and (c) 100 μg/ml drug. (d) Fluorescence images after AO/EB staining of B16-F10 cells at a concentration of 250 μg/ml of the drug on day 1, 3 and day 5 showing relative number cell density after treatment.

**(c)**

**Figure. S6.**

**
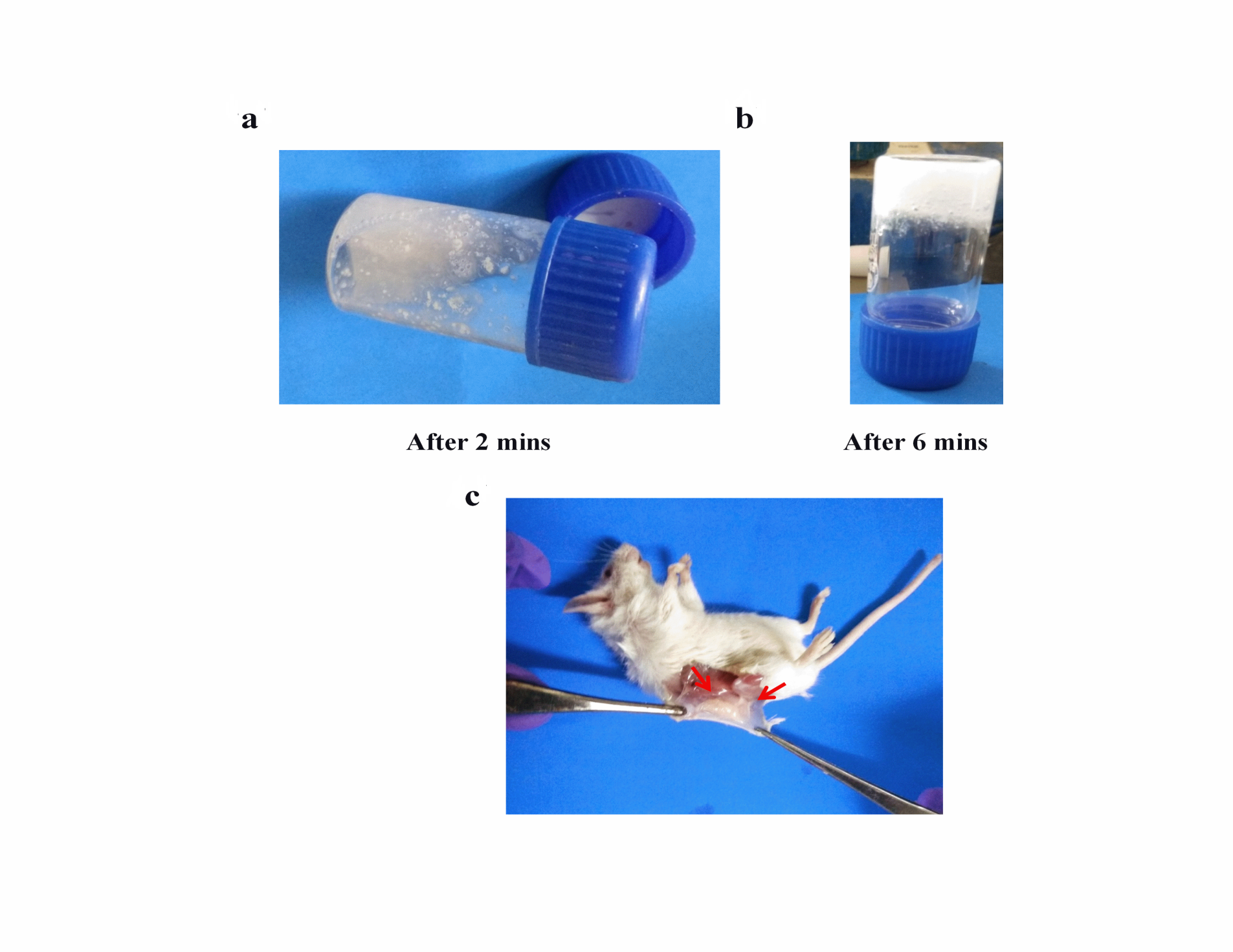
**

**Figure S6: In vitro and in vivo gelation digital images:** a) D-P-L-d was embedded in MC gel after 2 mins no gelation, (b) after 6 mins gel formed, and (c) in vivo images of injectable gel showing its integrity and localization at the injection site.

**(c)**

**Figure. S7.**


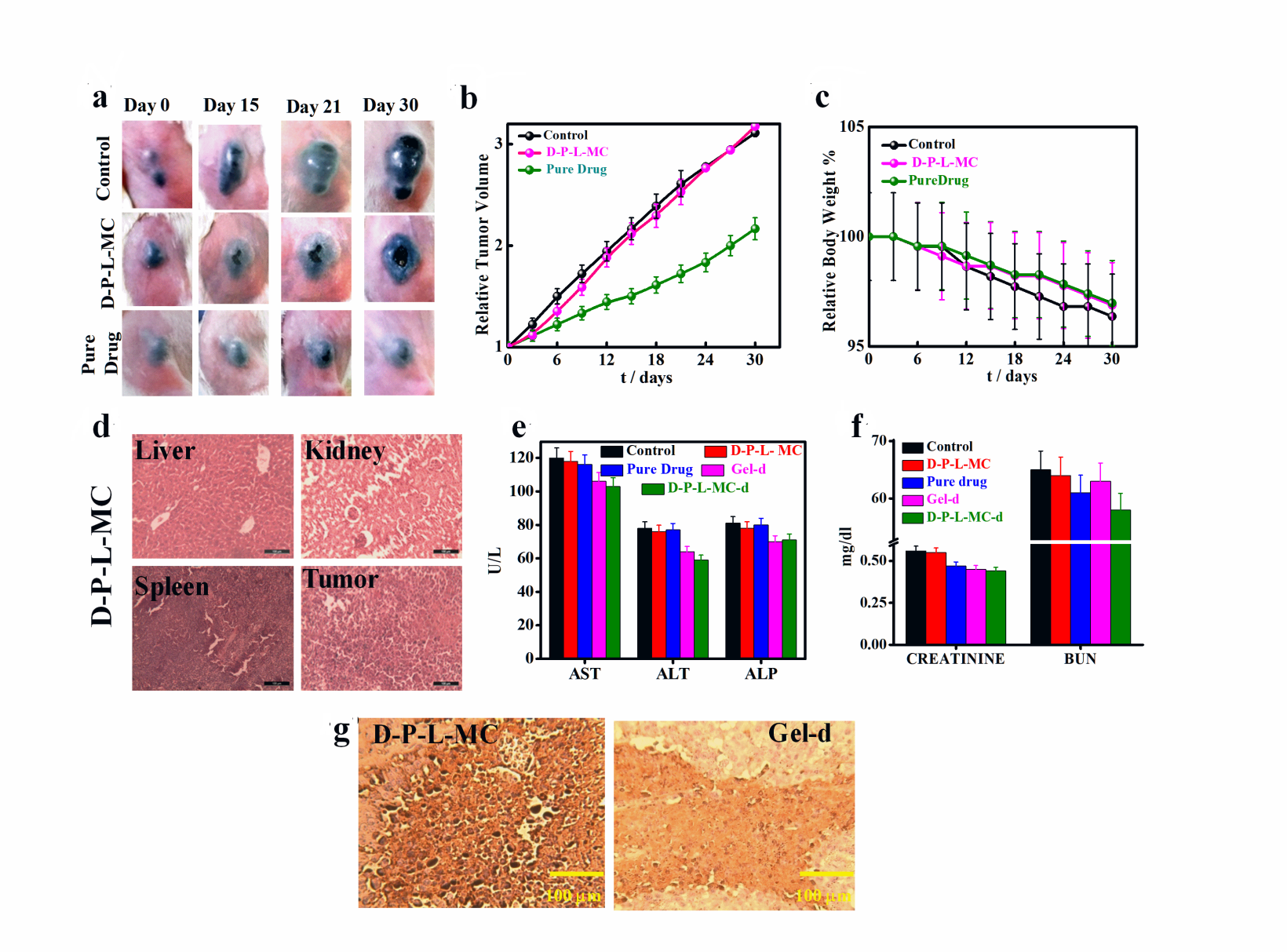


**Figure S7:** a) Images of mice after treatment with pure drug and pure polymer (D-P-L-MC), and control after different time interval. Tumor volume of control and D-P-L-MC treated mice increased with time, in case of pure drug slight suppression in tumor volume was observed, b) Relative tumor volume of pure polymer, drug and control showing slight suppression in tumor volume after treatment with drug as compared to other two systems. c) Relative body weight of control, pure drug and D-P-L-MC showing reduction in body weight after treatment with all the systems. d) Histopathological images of organs after treatment with pure polymer, indicating that number density of melanocytes was higher in tumor tissue. e) & f) Biochemical parameter analysis including AST, ALT, ALP, BUN and CREATININE. In all cases, the values of mice treated pure drug were elevated demonstrating significant damage in hepatic and renal systems due toxicity of drug. g) Immunostaining of tumor after treatment of 30 days with MIA showing overexpression in pure polymer and Gel-d signifying more melanoma cells.
